# Supplementary material for: A genomic lifespan program that reorganises the young adult brain is targeted in schizophrenia
Source: eLife. 2017 Sep 12;6:e17915. doi: 10.7554/eLife.17915 (PMC5595438; doi:10.7554/eLife.17915)
Supplement: Transparent reporting form [file elife-17915-transrepform.pdf]

eLIFE
1st Floor
24 Hills Road
Cambridge CB2 1JP, UK
P 01223 855340
W elifesciences.org
T @elife

eLife's transparent reporting form

We encourage authors to provide detailed information *within their submission* to facilitate the interpretation and replication of experiments. If you have any questions, please contact us: editorial@elifesciences.org.

Sample-size estimation

- You should state whether an appropriate sample size was computed when the study was being designed
- You should state the statistical method of sample size computation and any required assumptions
- If no explicit power analysis was used, you should describe how you decided what sample (replicate) size (number) to use

Please outline where this information can be found within the submission (e.g., page numbers or figure legends), or explain why this information doesn't apply to your submission:

The size of the two main transcriptome datasets used are stated on p6 lines 144 (Braincloud dataset) and line 152 (mouse hippocampus). Sample size is not itself the most important characteristic of the dataset and rather it is key that there be a good distribution across the lifespan: the distribution of ages is summarized in Supplementary Figure 2. We have performed no calculations for which a traditional power calculation for required sample size is relevant.

Replicates

- You should report how often each experiment was performed
- You should include a definition of biological versus technical replication
- The data obtained should be provided and sufficient information should be provided to indicate the number of independent biological and/or technical replicates
- If you encountered any outliers, you should describe how these were handled
- Criteria for exclusion/inclusion of data should be clearly stated
- High-throughput sequence data should be uploaded before submission, with a private link for reviewers provided (these are available from both GEO and ArrayExpress)

Please outline where this information can be found within the submission (e.g., page numbers or figure legends), or explain why this information doesn't apply to your submission:

There were no technical replicates in any of the datasets used. The mouse hippocampus data is available from ArrayExpress (E-MTAB-3256). All samples from all datasets were used, nor were any outliers dropped.

Statistical reporting

- Statistical analysis methods should be described and justified
- Raw data should be presented in figures whenever informative to do so (typically when N per group is less than 10)
- For each experiment, you should identify the statistical tests used, exact values of N, definitions of center, methods of multiple test correction, and dispersion and precision measures (e.g., mean, median, SD, SEM, confidence intervals; and, for the major substantive results, a measure of effect size (e.g., Pearson's r, Cohen's d)
- Report exact p-values wherever possible alongside the summary statistics and 95% confidence intervals. These should be reported for all key questions and not only when the p-value is less than 0.05.

Please outline where this information can be found within the submission (e.g., page numbers or figure legends), or explain why this information doesn't apply to your submission:

The primary statistical testing method used was bootstrapping. This is described between lines 714 – 874 and lines 907 – 986.

Line 148 describes the application of a Wilcoxon signed rank test to determine whether there is a gender difference in the age of turning points. The exact p-value is provided. Lines 741—746 in the methods section detail that the peak ages stated represent the mean age of turning points across all transcripts.

In line 154 we provide the p-value for the equivalent test performed in mice rather than humans. Wilcoxon signed rank test was again used as described in lines 741—746 in the methods section.

In lines 178—186 we describe the use of support vector machines to predict the age of humans and mice. The statistical methods underlying this are described in lines 966—986.

In lines 190—236 we apply Expression Weighted Celltype Enrichments (EWCE) to find cellular enrichments across the lifespan. This is a recently published method based on bootstrapping. We describe how it was done in the methods section lines 800—832. As stated in line 819 Bonferroni correction was used to correct all p-values. All p-values listed in the paper generated with EWCE have been Bonferroni corrected.

In line 248 we list a p-value associated with a gene ontology enrichment.

eLife Sciences Publications, Ltd is a limited liability non-profit non-stock corporation incorporated in the State of Delaware, USA, with company number 5030732, and is registered in the UK with company number FC030576 and branch number BR015634 at the address 1st Floor, 24 Hills Road, Cambridge CB2 1JP | August 2014

In lines 249—250 we list p-values associated with mammalian phenotype ontology (MPO) enrichments. In lines 254 we list p-values associated with enrichments in synaptic gene sets. MPO and synapse gene set enrichments were performed using hypergeometric tests. Multiple testing correction was not performed for MPO/synapse gene lists as only the targeted lists were tested. Bonferroni-Hochberg corrected p-value was used for the Gene Ontology enrichment. Further details are provided in methods lines 953—965.

In line 277 we mention that 99 of 900 proteins were found to have significant changes with age with p<0.005 after Bonferroni-Hochberg correction. Relevant statistics for each protein are provided in supplementary table 5. Analysis is described in methods lines 1028—1035.

In lines 310 and 313 we report two p-value obtained using the Wilcoxon rank sum method as described earlier as described in lines 741—746 in the methods section.

In lines 390 and 394 we list p-values obtained using hypergeometric tests. The expected and actual number of genes within tested genes set were listed along with the enrichment probability. The details are described in methods lines 1037—1055.

(For large datasets, or papers with a very large number of statistical tests, you may upload a single table file with tests, Ns, etc., with reference to page numbers in the manuscript.)

Additional data files ("source data")

- We encourage you to upload relevant additional data files, such as numerical data that are represented as a graph in a figure, or as a summary table
- Where provided, these should be in the most useful format, and they can be uploaded as "Source data" files linked to a main figure or table
- Include model definition files including the full list of parameters used
- Include code used for data analysis (e.g., R, MatLab)
- Avoid stating that data files are "available upon request"

Please indicate the figures or tables for which source data files have been provided:

Braincloud dataset is available online from http://braincloud.jhmi.edu/

The mouse hippocampus dataset is available through ArrayExpress (E-MTAB-3256).
